# Association between neutrophil to high-density lipoprotein cholesterol ratio and abdominal aortic calcification in US adults: A cross-sectional study

**DOI:** 10.1097/MD.0000000000049001

**Published:** 2026-05-22

**Authors:** Hailin Lu, Jiangfeng Zhang, Ming Hu, Zhong Qin, Xiao Qin, Han Yang, Jingpeng Wei

**Affiliations:** a Department of Vascular Surgery, The First Affiliated Hospital of Guangxi Medical University, Nanning, China; b Department of Vascular and Interventional Radiology, The First Affiliated Hospital of Guangxi University of Chinese Medicine, Nanning, China.

**Keywords:** abdominal aortic calcification, cross-sectional study, inflammation, National Health and Nutrition Examination Survey, neutrophil to high-density lipoprotein cholesterol ratio

## Abstract

Inflammation is involved in abdominal aortic calcification (AAC) pathogenesis. The neutrophil to high-density lipoprotein cholesterol ratio (NHR) is a novel index of inflammation assessment. However, little has been established about the relationship between NHR and AAC. This study aimed to investigate the connection between the NHR and AAC. Using data from the National Health and Nutrition Examination Survey conducted in 2013 to 2014, we analyzed the relationship between AAC score, severe AAC, and NHR through multiple linear and logistic regression models, along with restricted cubic spline (RCS) regression to assess the dose–response relationship. Subgroup analyses were conducted to find effect changes. Sensitivity analyses were performed to ensure study robustness. In fully adjusted model, elevated NHR positively associated with AAC score (β = 0.071, 95% CI: 0.006, 0.136) and severe AAC (OR = 1.094, 95% CI: 1.015, 1.176). Moreover, participants in the highest NHR quartile had an AAC score that was 0.637 units higher (β = 0.637, 95% CI: 0.335, 0.939) and an 69.8% higher risk of severe AAC (OR = 1.698, 95% CI: 1.152, 2.514). RCS showed the relationship between NHR and AAC score was nonlinear. However, the nonlinear relationship between the NHR and severe AAC was not significant. Subgroup analyses revealed no significant interactions regarding the correlation across stratification variable. Three sensitivity analyses demonstrated that the models in this research still yielded similar results. This research establishes a positive association between NHR and AAC prevalence. Our findings imply that NHR has the potential to serve as a biomarker of AAC.

## 1. Introduction

Abdominal aortic calcification (AAC) is a condition characterized by ectopic mineral deposition, primarily calcium phosphate, in abdominal aortic walls.^[1,2]^ To a certain degree, AAC can reflect the overall state of vascular calcification throughout the body,^[3]^ and serve as an indicator of elevated cardiovascular disease risk and cardiovascular mortality.^[4,5]^ Moreover, a prior study discovered that AAC was linked to cardiovascular disease mortality, and AAC showed a stronger association than coronary artery calcium with all-cause death.^[6]^ Therefore, early identification of AAC with simple, cost-effective, and convenient tools, is pressing.

In recent years, a wide range of immunoinflammatory markers, such as systemic immune inflammation index (SII), C-reactive protein and plasma atherogenic index, have been discovered and examined for their association with cardiovascular and vascular disease.^[7-11]^ Inflammation is also closely related to vascular calcification and hence may become a potential marker of AAC.^[12-14]^ In this sense, it would be crucial to have metrics that can reflect the individual “inflammatory state.” The neutrophil to high-density lipoprotein cholesterol ratio (NHR) is an emerging marker of both inflammation and lipid metabolism, reflecting the complex interaction between neutrophil cells activation and the protective effect of high-density lipoprotein (HDL).^[15,16]^ An increase in neutrophil count is associated with a systemic inflammatory response, while HDL is widely recognized for its anti-inflammatory properties.^[17,18]^ In several recent studies, the NHR has shown potential as a biomarker for inflammation and cardiovascular risk.^[19-21]^ While these findings highlight NHR’s sensitivity, they raise intriguing possibilities about its specificity for diagnosing AAC, which remains limited. Therefore, exploring the relationship between NHR and AAC holds practical clinical significance.

This analysis aims to explore the potential of NHR as a biomarker within the AAC. We tested the conjecture that an elevated NHR is correlated with an increased likelihood of having AAC by using 2013 to 2014 National Health and Nutrition Examination Survey (NHANES) database.

## 2. Materials and methods

### 2.1. Study population

**Figure 1.**
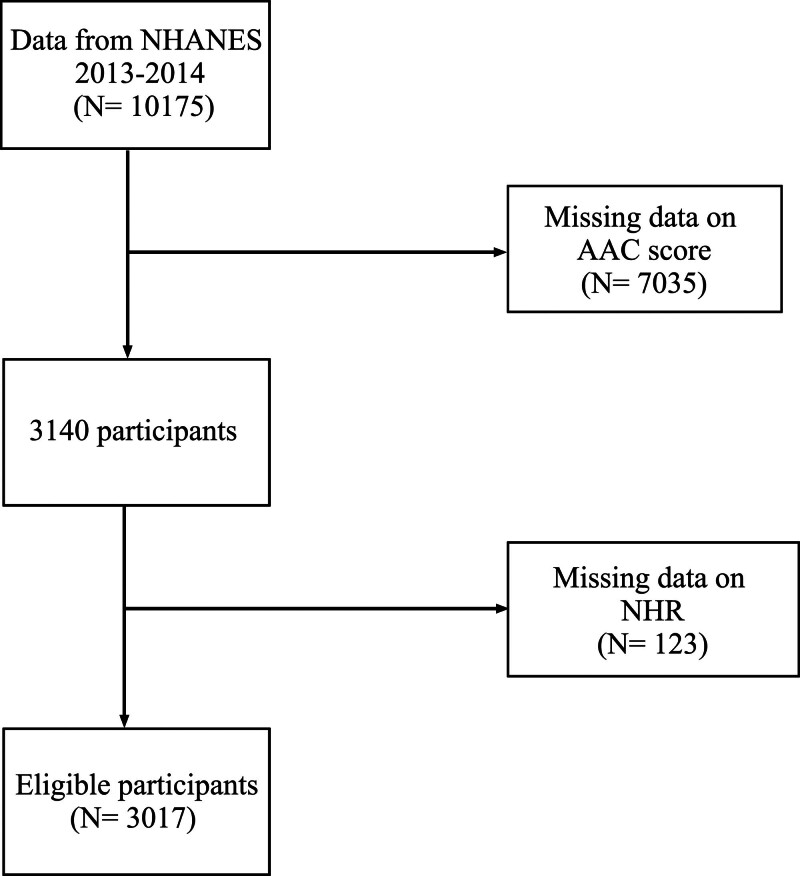
Flowchart of participant recruitment. AAC = abdominal aortic calcification, NHANES = National Health and Nutrition Examination Survey, NHR = neutrophil/high-density lipoprotein cholesterol ratio.

This cross-sectional study initially included participants from NHANES 2013–2014. Demographics, examinations, laboratory tests, and questionnaires data of participants were collected from the NHANES database. After the exclusion of those with AAC missing (n = 7035) and those with NHR missing (n = 123), a total of 3017 participants were finally included (Fig. 1). All participants provided written informed consent prior to their enrollment in NHANES. The protocol for this research was reviewed and approved by the Research Ethics Review Committee of the National Center for Health Statistics.

### 2.2. Measurement of NHR

Neutrophil to high-density lipoprotein cholesterol ratio was calculated as the neutrophil count (10^3^ cells/mL) divided by HDL-C concentration (mmol/L).^[22]^ Both the numerator and denominator of NHR are acquired via laboratory testing. The absolute number of neutrophils is determined using the UniCel DxH 800 analyzer (Beckman Coulter, Inc.). HDL cholesterol levels are precisely measured on Roche Modular P and Roche Cobas 6000 (Roche Diagnostics) chemistry analyzers. These processes align with the standardized protocols specified in the NHANES.

### 2.3. Assessment of AAC

The AAC score was derived from examination data collected by NHANES. Trained and certified radiologic technologists assessed AAC load in the lateral lumbar spine region (vertebrae L1–L4) using dual-energy x-ray absorptiometry (DXA). AAC scores were then calculated employing the Kauppila scoring system. A comprehensive description of the protocol is available in the NHANES “Body Composition Procedures Manual.” In view of the previous research,^[23,24]^ severe AAC in this study was defined as >6 score.

### 2.4. Covariates

Potential confounders were identified through literature review and encompassed demographic variables (age, gender, race, education level, and poverty income ratio [PIR]), lifestyle factors (alcohol consumption [participants who had at least 12 alcohol drinks annually] and smoking [participants who having smoked a minimum of 100 cigarettes throughout their life]), physical examination data (waist circumference and body mass index [BMI]), biochemical markers (triglycerides [TG], total cholesterol [TC], HDL-C, serum calcium, serum phosphorus, total 25-hydroxyvitamin D), and comorbidities (hypertension, hypercholesterolemia, diabetes, coronary heart disease, stroke, chronic obstructive pulmonary disease [COPD], malignancy). Definitions and detailed information for each covariate are provided in Table S1.

### 2.5. Statistical analyses

Categorical variables were expressed as frequencies and percentages and continuous variables reported as median (25th to 75th percentile). Comparison of general characteristics stratified by AAC status and NHR-group were performed using the Wilcoxon rank sum test, Fisher exact test and Pearson chi-squared test for trend as appropriate to data distribution.

Multiple linear and logistic regression models were used to explore the independent relationship between NHR and AAC (including AAC score and severe AAC) in 3 different models. In the crude model (model 1), no covariate was adjusted. In the partially adjusted model (model 2), age and race were included. In the fully adjusted model (model 3), age, race, BMI, diabetes, hypercholesterolemia, hypertension, smoke, coronary heart disease, stroke, COPD, malignancy, serum phosphorus, total 25-hydroxyvitamin D, and total cholesterol were adjusted. Additionally, restricted cubic spline regression (3 knots) was employed to investigate underlying nonlinear associations between NHR and AAC. The nonlinearity was assessed with the likelihood ratio test. Stratified analyses were conducted to assess effect modification by variables including age, race, smoking status, and the presence of hypertension, hypercholesterolemia, and diabetes. All covariates were adjusted except for the stratification variable itself. Interaction tests were conducted by incorporating interaction terms between NHR and each stratification variable into the regression models, and interaction *P*-values were reported to evaluate subgroup differences. Three sensitivity analyses were performed to ensure study robustness. Sensitivity analysis 1 was conducted by complementing the missing values. Sensitivity analysis 2 was performed by excluding participants with extreme values of NHR and other continuous covariates. Sensitivity analysis 3 was undertaken by analyzing participants aged ≥60 years.

All analysis was conducted using the SPSS software (version 24.0, IBM Corp.) and the R software (version 4.3.0, R Foundation for Statistical Computing). A 2-sided *P*-value < .05 was considered statistically significant.

## 3. Results

### 3.1. Baseline characteristics

The present study finally included 3017 individuals (Fig. 1), with a median age of 58.0 years, 48% (1455) of whom were male. Severe AAC was present in 270 (8.9%) participants, and the median NHR was 2.95. General characteristics of the study population, stratified by NHR tertiles (T1, 0.35–2.39; T2, 2.40–3.75; T3, 3.76–21.58) and severe AAC status are shown in Tables 1 and 2, respectively. Significant differences in gender, race, education levels, BMI, PIR, waist circumference, smoking and drinking status, hypertension, hypercholesterolemia, diabetes, coronary heart disease, COPD, serum phosphorus, total 25-hydroxyvitamin D, triglycerides, total cholesterol, HDL and AAC scores were observed across NHR tertiles. Serum phosphorus, total 25-hydroxyvitamin D were higher in individuals with severe AAC than in those without severe AAC, but the participants in severe AAC group had lower levels of BMI and total cholesterol. Moreover, participants with severe AAC were more likely to be older, smokers, non-Hispanic White and tend to have hypertension, diabetes, hypercholesterolemia, coronary heart disease, stroke, COPD and malignancy, compared to those without severe AAC. Besides, participants exhibiting severe AAC presented with elevated NHR scores, achieving statistical significance (*P *< .01). No significant differences were observed in gender, education level, PIR, waist circumference, alcohol consumption, serum calcium, triglycerides, and HDL (*P > *.05).

**Table 1:** Baseline characteristics of patients stratified by NHR.

Characteristic	Overall	Tertile 1 (0.35–2.39) N = 1006	Tertile 2 (2.40–3.75) N = 1003	Tertile 3 (3.76–21.58) N = 1008	*P* value
Age, yr	58 (48, 68)	59 (49, 68)	57 (48, 68)	57 (47, 68)	.157
Age strata, n (%)					.356
<60	1612 (53%)	519 (52%)	544 (54%)	549 (54%)	
≥60	1405 (47%)	487 (48%)	459 (46%)	459 (46%)	
Gender, n (%)					**<.001**
Male	1455 (48%)	361 (36%)	516 (51%)	578 (57%)	
Female	1562 (52%)	645 (64%)	487 (49%)	430 (43%)	
Race, n (%)					**<.001**
Mexican American	401 (13%)	82 (8%)	158 (16%)	161 (16%)	
Other Hispanic	285 (10%)	81 (8%)	104 (10%)	100 (10%)	
Non-Hispanic White	1340 (44%)	393 (39%)	435 (43%)	512 (51%)	
Non-Hispanic Black	576 (19%)	291 (29%)	165 (16%)	120 (12%)	
Other race	415 (14%)	159 (16%)	141 (14%)	115 (11%)	
Education, n (%)					**<.001**
<High school	689 (23%)	198 (20%)	228 (23%)	263 (26%)	
High school	685 (23%)	204 (20%)	221 (22%)	260 (26%)	
>High school	1643 (54%)	604 (60%)	554 (55%)	485 (48%)	
PIR	2.39 (1.17, 4.35)	2.84 (1.25, 4.84)	2.52 (1.23, 4.54)	2.05 (1.07, 3.82)	**<.001**
BMI, kg/m^2^	27.8 (24.5, 31.7)	26.0 (22.8, 29.8)	27.8 (24.8, 31.5)	29.6 (26.3, 33.5)	**<.001**
Waist circumference, cm	99 (90, 108)	93 (85, 102)	99 (91, 107)	104 (96, 114)	**<.001**
Smoke, n (%)					**<.001**
Yes	1393 (46%)	400 (40%)	430 (43%)	563 (56%)	
No	1624 (54%)	606 (60%)	573 (57%)	445 (44%)	
Alcohol, n (%)					**.006**
Yes	2029 (67%)	638 (63.4%)	695 (69.3%)	696 (69%)	
No	817 (27%)	304 (30.2%)	258 (25.7%)	255 (25.3%)	
Missing	171 (6%)	64 (6.4%)	50 (5%)	57 (5.7%)	
Diabetes, n (%)					**<.001**
Yes	661 (22%)	126 (13%)	208 (21%)	327 (32%)	
No	2356 (78%)	880 (87%)	795 (79%)	681 (68%)	
Hypercholesterolemia, n (%)					**<.001**
Yes	1503 (50%)	438 (44%)	515 (51%)	550 (55%)	
No	1514 (50%)	568 (56%)	488 (49%)	458 (45%)	
Hypertension, n (%)					**<.001**
Yes	1428 (47%)	427 (42%)	464 (46%)	537 (53%)	
No	1589 (53%)	579 (58%)	539 (54%)	471 (47%)	
Coronary heart disease, n (%)					**<.001**
Yes	158 (5%)	27 (2.7%)	55 (5.5%)	76 (7.5%)	
No	2859 (95%)	979 (97.3%)	948 (94.5%)	932 (92.5%)	
Stroke, n (%)					.190
Yes	130 (4%)	38 (3.8%)	39 (3.9%)	53 (5.3%)	
No	2887 (96%)	968 (96.2%)	964 (96.1%)	955 (94.7%)	
COPD, n (%)					**<.001**
Yes	127 (4%)	27 (2.7%)	34 (3.4%)	66 (6.5%)	
No	2890 (96%)	979 (97.3%)	969 (96.6%)	942 (92.5%)	
Malignancy, n (%)					.482
Yes	375 (12%)	131 (13%)	129 (12.9%)	115 (11.4%)	
No	2642 (88%)	875 (87%)	874 (87.1%)	893 (88.6%)	
Serum calcium, mmol/L	9.40 (9.20, 9.70)	9.40 (9.20, 9.70)	9.40 (9.20, 9.60)	9.40 (9.20, 9.70)	.227
Serum phosphorus, mmol/L	1.23 (1.10, 1.36)	1.23 (1.13, 1.36)	1.23 (1.10, 1.32)	1.20 (1.07, 1.32)	**.004**
Triglycerides, mg/dL	100 (68, 149)	79 (57, 111)	102 (76, 149)	134 (93, 201)	**<.001**
HDL-C, mg/dL	51 (42, 63)	65 (55, 78)	51 (45, 59)	41 (35, 48)	**<.001**
Total cholesterol, mg/dL	193 (166, 220)	200 (175, 227)	191 (166, 217)	186 (159, 217)	**<.001**
Total 25-hydroxyvitamin D, nmol/L	67 (51, 86)	70 (51, 91)	67 (51, 85)	65 (50, 82)	**<.001**
AAC score	0 (0, 2)	0 (0, 1)	0 (0, 2)	0 (0, 2)	**<.001**
Severe AAC					**<.001**
No	2747 (91%)	940 (93%)	914 (91%)	893 (89%)	
Yes	270 (8.9%)	66 (6.6%)	89 (8.9%)	115 (11%)	

Bold values indicate statistical significance.

AAC = abdominal aortic calcification, BMI = body mass index, COPD = chronic obstructive pulmonary disease, HDL-C = high-density lipoprotein cholesterol, NHR = neutrophil to high-density lipoprotein cholesterol ratio, PIR = poverty-to-income ratio.

**Table 2:** Baseline characteristics of subjects with and without severe AAC.

Characteristics	Overall	Severe AAC		*P* value
	N = 3017	No = 2747	Yes = 270	
Age, yr	58 (48, 68)	56 (47, 66)	74 (65, 80)	**<.001**
Age strata, n (%)				**<.001**
<60	1612 (53%)	1580 (58%)	32 (12%)	
≥60	1405 (47%)	1167 (42%)	238 (88%)	
Gender, n (%)				.877
Male	1455 (48%)	1326 (48%)	129 (48%)	
Female	1562 (52%)	1421 (52%)	141 (52%)	
Race, n (%)				**<.001**
Mexican American	401 (13.3%)	378 (13.8%)	23 (8.5%)	
Other Hispanic	285 (9.4%)	274 (10.0%)	11 (4.1%)	
Non-Hispanic White	1340 (44.4%)	1164 (42.4%)	176 (65.2%)	
Non-Hispanic Black	576 (19.1%)	542 (19.7%)	34 (12.6%)	
Other race	415 (13.8%)	389 (14.2%)	26 (9.6%)	
Education, n (%)				.234
<High school	689 (23%)	619 (23%)	70 (26%)	
High school	685 (23%)	619 (23%)	66 (24%)	
>High school	1643 (54%)	1509 (55%)	134 (50%)	
PIR	2.39 (1.17, 4.35)	2.42 (1.17, 4.44)	2.12 (1.20, 3.83)	.241
BMI, kg/m^2^	27.8 (24.5, 31.7)	27.9 (24.5, 31.9)	26.6 (24.2, 29.9)	**<.001**
Waist circumference, cm	99 (90, 108)	99 (90, 108)	98 (92, 105)	.478
Smoke, n (%)				**<.001**
Yes	1393 (46%)	1229 (45%)	164 (61%)	
No	1624 (54%)	1518 (55%)	106 (39%)	
Alcohol, n (%)				.131
Yes	2029 (67%)	1842 (67%)	187 (69%)	
No	817 (27%)	742 (27%)	75 (28%)	
Missing	171 (6%)	163 (6%)	8 (3%)	
Diabetes, n (%)				**<.001**
Yes	661 (22%)	559 (20%)	102 (38%)	
No	2356 (78%)	2188 (80%)	168 (62%)	
Hypercholesterolemia, n (%)				**<.001**
Yes	1503 (50%)	1310 (48%)	193 (71%)	
No	1514 (50%)	1437 (52%)	77 (29%)	
Hypertension, n (%)				**<.001**
Yes	1428 (47%)	1226 (45%)	202 (75%)	
No	1589 (53%)	1521 (55%)	68 (25%)	
Coronary heart disease, n (%)				**<.001**
Yes	158 (5%)	104 (3.8%)	54 (20%)	
No	2859 (95%)	2643 (96.2%)	216 (80%)	
Stroke, n (%)				**<.001**
Yes	130 (4%)	99 (3.6%)	31 (11.5%)	
No	2887 (96%)	2648 (96.4%)	239 (88.5%)	
COPD, n (%)				**<.001**
Yes	127 (4%)	103 (3.7%)	24 (8.9%)	
No	2890 (96%)	2644 (96.3%)	246 (91.1%)	
Malignancy, n (%)				**<.001**
Yes	375 (12%)	304 (11.1%)	71 (26.3%)	
No	2642 (88%)	2443 (88.9%)	199 (73.7%)	
Serum calcium, mmol/L	2.35 (2.30, 2.43)	2.35 (2.30, 2.43)	2.38 (2.30, 2.43)	.123
Serum phosphorus, mmol/L	1.23 (1.10, 1.36)	1.23 (1.10, 1.36)	1.26 (1.13, 1.36)	**.041**
Triglycerides, mg/dL	100 (68, 149)	100 (68, 149)	107 (77, 150)	.297
HDL-C, mg/dL	51 (42, 63)	51 (43, 63)	50 (41, 64)	.229
Total 25-hydroxyvitamin D, nmol/L	67 (51, 86)	66 (50, 85)	79 (60, 97)	**<.001**
Total cholesterol, mg/dL	193 (166, 220)	194 (167, 221)	176 (155, 208)	**<.001**
AAC score	0 (0, 2)	0 (0, 0)	10 (8, 14)	**<.001**
NHR	2.95 (2.09, 4.18)	2.93 (2.07, 4.14)	3.42 (2.40, 4.66)	**<.001**
NHR group, n (%)				**.001**
Tertile 1	1006 (33%)	940 (34%)	66 (24%)	
Tertile 2	1003 (33%)	914 (33%)	89 (33%)	
Tertile 3	1008 (33%)	893 (33%)	115 (43%)	

AAC = abdominal aortic calcification, BMI = body mass index, COPD = chronic obstructive pulmonary disease, HDL-C = high-density lipoprotein cholesterol, NHR = neutrophil to high-density lipoprotein cholesterol ratio, PIR = poverty-to-income ratio.

### 3.2. Relationship between NHR and AAC

Table 3 shows multiple linear and logistic regression models that illustrate the association between NHR and AAC. In the crude model (model 1), no covariates were adjusted. Partially adjusted model (model 2) was adjusted for age and race. Both the model 1 and model 2 showed a positive relationship between NHR and AAC score (β = 0.102, 95% CI: 0.036, 0.168, *P* = .003 for model 1; β = 0.118, 95% CI: 0.056, 0.180, *P < *.001 for model 2). Fully adjusted model (model 3) was adjusted for age, race, BMI, diabetes, hyperlipidemia, hypertension, smoke, coronary heart disease, stroke, COPD, malignancy, serum phosphorus, total 25-hydroxyvitamin D, and total cholesterol. The positive association was still stable in model 3 (β = 0.071, 95% CI: 0.006, 0.136, *P* = .033), indicating that each 1 unit of increased NHR was associated with 0.071 increased unit of AAC score. We also converted NHR from a continuous variable to a categorical variable (tertiles). AAC score increased with the higher NHR tertiles group (*P* for trend < .001 in model 1, *P* for trend < .001 in model 2). In the fully adjusted model (model 3), the AAC score of the highest NHR tertile (tertile 3) was 0.637 units higher compared with the lowest tertile (β = 0.637, 95% CI: 0.335, 0.939, *P* < .001).

**Table 3:** Relationship between NHR, AAC score, and severe AAC.

Exposure	AAC score		Severe AAC	
	β (95% CI)	*P* value	OR (95% CI)	*P* value
Crude model (model 1)*				
Continuous NHR	0.102 (0.036, 0.168)	**.003**	1.098 (1.036, 1.161)	**.001**
Categories NHR				
Tertile 1	Reference	–	Reference	–
Tertile 2	0.354 (0.048, 0.660)	**.024**	1.387 (0.998, 1.937)	.053
Tertile 3	0.709 (0.403, 1.015)	**<.001**	1.834 (1.341, 2.527)	**<.001**
*P* for trend	0.501 (0.285, 0.717)	**<.001**	1.536 (1.231, 1.926)	**<.001**
Partially adjusted model (model 2)†				
Continuous NHR	0.118 (0.056, 0.180)	**<.001**	1.136 (1.063, 1.212)	**<.001**
Categories NHR				
Tertile 1	Reference	–	Reference	–
Tertile 2	0.392 (0.109, 0.674)	**.007**	1.394 (0.977, 1.998)	.068
Tertile 3	0.762 (0.477, 1.047)	**<.001**	1.962 (1.394, 2.781)	**<.001**
*P* for trend	0.539 (0.337, 0.741)	**<.001**	1.611 (1.265, 2.061)	**<.001**
Fully adjusted model (model 3)‡				
Continuous NHR	0.071 (0.006, 0.136)	**.033**	1.094 (1.015, 1.176)	**.015**
Categories NHR				
Tertile 1	Reference	–	Reference	–
Tertile 2	0.328 (0.045, 0.611)	**.023**	1.337 (0.914, 1.963)	.136
Tertile 3	0.637 (0.335, 0.939)	**<.001**	1.698 (1.152, 2.514)	**.008**
*P* for trend	0.450 (0.237, 0.664)	**<.001**	1.454 (1.106, 1.919)	**.008**

Bold values indicate statistical significance.

AAC = abdominal aortic calcification, BMI = body mass index, CI = confidence interval, COPD = chronic obstructive pulmonary disease, NHR = neutrophil to high-density lipoprotein cholesterol ratio, OR = odds ratio.

* Crude model (model 1): no covariates were adjusted.

† Partially adjusted model (model 2): adjusted for age and race.

‡ Fully adjusted model (model 3): age, race, BMI, diabetes, hypercholesterolemia, hypertension, smoke, coronary heart disease, stroke, COPD, malignancy, serum phosphorus, total 25-hydroxyvitamin D, and total cholesterol were adjusted.

Table 3 also displays the regression models of severe AAC with NHR. The risk of severe AAC increased as NHR rose in both the model 1 and model 2. Specifically, in model 3, the result indicated that each 1 unit of increased NHR was associated with a 109.4% increased risk of severe AAC (95% CI: 1.015, 1.176, *P* = .015). Meanwhile, the risk of severe AAC increased with the highest NHR tertiles (model 1, OR = 1.834, 95% CI: 1.341, 2.527, *P* < .001, *P* for trend < .001; model 2, OR = 1.962, 95% CI: 1.394, 2.781, *P* < .001, *P* for trend < .001; model 3, OR = 1.698, 95% CI: 1.152, 2.514, *P* = .008, *P* for trend = .008) compared with the lowest tertile.

### 3.3. Subgroup and sensitivity analysis

**Figure 2.**
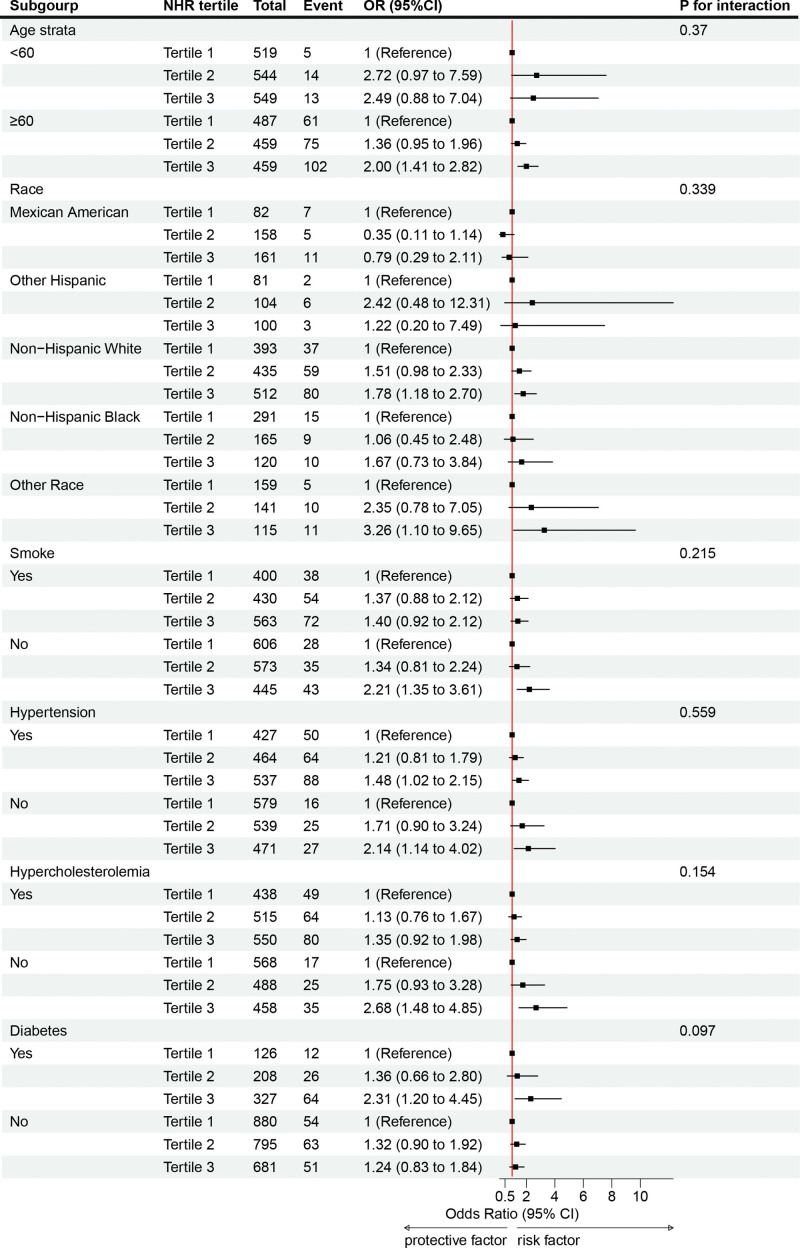
Forest plot of the relationship between NHR and severe AAC in different subgroups. AAC = abdominal aortic calcification, CI = confidence interval, NHR = neutrophil/high-density lipoprotein cholesterol ratio, OR = odds ratio.

The subgroup analysis showed that the link between NHR categories and severe AAC exhibited consistency within the following subgroups: age, race, smoke, hypertension, hypercholesterolemia, and diabetes (Fig. 2). Sensitivity analyses demonstrated that the models in this research still yielded similar results after multiple imputation for missing data, exclusion of participants with extreme values of NHR and other continuous covariates, and restriction to participants aged ≥60 years (Tables S2–S4, respectively).

### 3.4. Dose-relationship between NHR and AAC

**Figure 3.**
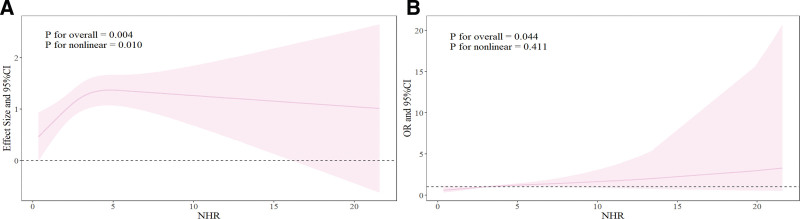
RCS curve of the association between NHR and AAC. (A) The association between NHR and AAC score; (B) the association between NHR and severe AAC. Age, race, BMI, diabetes, hypercholesterolemia, hypertension, smoke, coronary heart disease, stroke, COPD, malignancy, serum phosphorus, total 25-hydroxyvitamin D, and total cholesterol were adjusted. AAC = abdominal aortic calcification, BMI = body mass index, CI = confidence intervals, COPD = chronic obstructive pulmonary disease, NHR = neutrophil to high-density lipoprotein cholesterol ratio, OR = odds ratios, RCS = restricted cubic spline.

As shown in Figure 3A, the dose–response analysis with a restricted cubic spline model showed a significant nonlinear relationship between NHR and the AAC score (*P* for nonlinearity = .001). However, the nonlinear relationship between the NHR and severe AAC, as shown in Figure 3B, was not significant (*P* for nonlinearity = .411), suggesting that the relationship was a near-linear one. Of course, to confirm these findings, more extensive prospective studies are needed to thoroughly validate the role of NHR in the development of AAC.

## 4. Discussion

This study uncovered the relationship between NHR and AAC by analyzing real-world sample data from the NHANES database. Our research revealed an association between the NHR level and the likelihood of experiencing AAC, which persisted even after adjusting for other factors. Subgroup analyses further confirmed that this relationship was maintained, regardless of age, race, exposure to tobacco and history of hypertension, hypercholesterolemia, and diabetes. Sensitivity analyses demonstrated the robustness of this association. The dose–response analysis showed a gradual increase in AAC risk with higher NHR. These findings indicate that NHR may function as a biological marker to assess the risk of developing AAC.

Extensive research has demonstrated that NHR has the capacity to predict multiple diseases, such as depression, gallstones, diabetes, and chronic kidney disease.^[25-28]^ However, the correlation between NHR and AAC has not been reported. Although former research has not specifically focused on the relationship between NHR and AAC, recent investigations have explored the association between AAC and other hematological markers, particularly those related to neutrophils and HDL-C. Current study provides robust evidence supporting the notion that inflammation is fundamental to the pathogenesis of vascular calcification.^[29,30]^ This is primarily attributed to the inflammation’s regulation of vascular smooth muscle cell expression, a process that promotes calcification.^[31]^ Especially, neutrophils are crucial in the formation of calcified plaques. They oxidize and degrade lipids and proteins via the release of oxidative stress product and proteases, leading to inflammation and calcium deposition in the arterial wall.^[32]^ According to recent research by Deng et al^[33]^, the neutrophil-to-lymphocyte ratio (NLR) exhibited a positive correlation with severe AAC, even after accounting for potential confounders. A multicenter research conducted by Ban et al revealed a significant association between NLR and AAC in patients with end-stage renal disease.^[34]^ Cheng et al reported several neutrophils related systemic immunoinflammatory biomarkers are linked to severe AAC.^[35]^ The combination of those clinical biomarkers not only helps to develop models for predicting severe AAC, but also may assist in early diagnosis and personalized treatment.

In regard to the relationship between AAC and HDL-related inflammation index. Chen et al carried out a retrospective dual-center study to investigate the cross-sectional relationship between platelet to HDL-C ratio (PHR) and AAC in maintenance hemodialysis patients.^[36]^ Through a rigorous investigation, they discovered a linkage between lower HDL-C concentrations and an amplified susceptibility to AAC. PHR was independently associated with the presence of AAC and showed the strongest correlation with AAC among the 3 selected markers. Furthermore, Ding et al investigated a novel inflammatory marker, the monocyte-to-high-density lipoprotein cholesterol ratio (MHR), in a representative sample of 2379 individuals using NHANES data.^[37]^ Through their investigation, they revealed a marked association between raised MHR values and an increased risk of AAC. A cross-sectional study analyzed data from 2789 US adults found participants in the highest uric acid to high-density lipoprotein cholesterol ratio (UHR) quartile faced a 122.7% greater risk of severe AAC compared to the lowest quartile, suggesting that UHR could be a potential clinical marker for AAC.^[38]^ Our study revealed that each unit increase in NHR might increase the risk of severe AAC by 9.4%. Furthermore, compared to the lowest NHR group (tertile 1), the highest NHR group (tertile 3) might have a 69.8% higher risk of severe AAC. These results align with previous researches, suggesting that excessively high NHR levels are linked to an increased risk of severe AAC.

Despite the unclear mechanism linking NHR and AAC, it was possible that inflammation and oxidative stress significantly contributed to their relationship. When NHR is elevated, it may be attributed to an increase in neutrophil counts, leading to the release of substantial amounts of inflammatory mediators and reactive oxygen species that induces increased oxidative stress, ultimately resulting in endothelial cell dysfunction and structural and functional deterioration of the cardiovascular system.^[39]^ Given this, modulating inflammatory responses and controlling redox signaling is essential for maintaining endothelial function and preserving vascular homeostasis.^[40]^ Furthermore, elevated NHR may also be due to a reduction in HDL-C concentrations, as low HDL-C concentrations weaken the inhibitory effect of endothelial cell inflammation. HDL-C could engage with endothelial cell membranes, then decreasing their activation and the expression of inflammatory mediators.^[41]^ This reduction in inflammation can subsequently mitigate the negative effects of inflammatory cytokines on the abdominal aortic walls, thereby modulating calcium phosphate disturbances linked to abdominal aortic walls. Besides, low HDL-C concentrations attenuated the capacity of removing oxidized cholesterol from endothelial cells. The accumulation of lipids promotes atherosclerosis, results in endothelial dysfunction, ultimately influencing the progression of vascular calcification.^[42,43]^ Even more, neutrophils can erode the antioxidant and anti-inflammatory functions of HDL-C and accelerate the oxidation of LDL-C via degranulation.^[44,45]^ Therefore, combining the above effects of neutrophil and HDL-C, NHR could increase the burden of inflammation and oxidative stress, and thus predict AAC risk. Collectively, these results support the “neutrophil activation–oxidative stress–HDL dysfunction” axis as a biologically plausible pathway that underlies the link between NHR and AAC pathology.

### 4.1. Strengths and limitations

This study presents several significant advantages. Particularly, a nationally representative sample that mirrors the demographic variety of US adults is employed, making it noteworthy that it leads the investigation of the association between the NHR and the risk of AAC. This large and diverse sample base strengthens the external validity of the findings. Furthermore, the robustness of the present study is enhanced by rigorous subgroup analyses and the adjustment of key covariates, which substantiate the reliability of the conclusions drawn herein. Moreover, the nonlinear relationship between NHR and AAC was further investigated, thereby yielding a more nuanced insight into the complex interplay among these variables. Ultimately, to the best of our knowledge, no study has evaluated the association of NHR with AAC. Drawing on existing data, this research represents a pioneering effort to explore the correlation between NHR and AAC.

However, there are certain limitations associated with this study. First, the cross-sectional design cannot establish causality, given that it offers a single-timepoint assessment instead of a longitudinal perspective. Therefore, the conclusions of our study should be interpreted with caution. Second, relying on self-reported questionnaires to estimate health status creates the risk of recall and response bias. Third, this research does not elucidate the underlying molecular mechanisms linking the observed NHR to AAC risk, thereby limiting our understanding of the causal relationship. Fourth, the exclusive focus on NHR as a putative biomarker for AAC carries the risk of overlooking other significant biomarkers or combinations of markers (like the SII and NLR) that could provide a more holistic assessment of the risk profile for AAC. Consequently, without a comparative analysis of the predictive performance of NHR with these common inflammatory indices may limit the strength of this study. Fifth, there remains a risk that residual or unmeasured confounders, such as renal failure, dietary supplement, and the use of antihyperlipidemic medications, could potentially bias the results,^[46,47]^ thereby affecting the interpretation of the findings. Hence, a cautious approach is warranted when interpreting our findings, and further validation under different conditions is required. Sixth, while a significant nonlinear relationship was identified, the clinical implications of this finding require further exploration. Last, because NHANES participants were sampled from the US population, the findings may not be directly generalized to other countries or ethnic groups.

## 5. Conclusion

In this study, we found that NHR was associated with the prevalence of AAC. Our findings hint that NHR has the potential to serve as a biomarker of AAC. Of course, to confirm these findings, future prospective, multicenter researches are needed to thoroughly validate the role of NHR in the development of AAC.

## Acknowledgments

We sincerely thank the NHANES platform and all participants in this study.

## Author contributions

**Conceptualization:** Jingpeng Wei.

**Data curation:** Ming Hu, Zhong Qin.

**Formal analysis:** Jiangfeng Zhang, Ming Hu, Zhong Qin.

**Funding acquisition:** Hailin Lu.

**Investigation:** Hailin Lu.

**Methodology:** Hailin Lu, Han Yang, Jingpeng Wei.

**Project administration:** Xiao Qin, Jingpeng Wei.

**Resources:** Xiao Qin, Han Yang.

**Software:** Hailin Lu, Han Yang, Jingpeng Wei.

**Supervision:** Xiao Qin.

**Validation:** Hailin Lu, Jiangfeng Zhang, Ming Hu, Jingpeng Wei.

**Writing – original draft:** Hailin Lu, Jiangfeng Zhang, Ming Hu.

**Writing – review & editing:** Jingpeng Wei.








