# Supplementary material for: Association between neutrophil to high-density lipoprotein cholesterol ratio and abdominal aortic calcification in US adults: A cross-sectional study
Source: Medicine (Baltimore). 2026 May 22;105(21):e49001. doi: 10.1097/MD.0000000000049001 (PMC13200935; doi:10.1097/MD.0000000000049001)
Supplement: Supplementary file 1 [file medi-105-e49001-s001.docx]

**S1 Table** Covariates and categorical definition of some of them.

|  | **Covariates** | **Definition** |
| --- | --- | --- |
| **Demographic variables** | age strata | < 60 years and ≥ 60 years |
|  | gender | Male and female |
|  | race | Mexican American, Other Hispanic, On-Hispanic White, On-Hispanic Black, Other Race |
|  | poverty income ratio (PIR) | \ |
|  | education level | < high school, high school or equivalent, >high school |
|  |  |  |
| **Examination variables** | body mass index (BMI) ^2^ | \ |
|  | waist circumference (cm) | \ |
|  |  |  |
| **Laboratory variables** | total cholesterol (mg/dL) | \ |
|  | triglycerides (mg/dL) | \ |
|  | high-density lipoprotein cholesterol  (HDL-C, mg/dL) | \ |
|  | total 25-hydroxyvitamin D (nmol/L) | \ |
|  | serum calcium (mmol/L) | \ |
|  | serum phosphorus (mmol/L) | \ |
|  | neutrophil count | \ |
|  |  |  |
| **Living health variables** | smoking status | Yes (having smoked a minimum of 100 cigarettes throughout their life) |
|  | alcohol consumption | Yes (minimum of 12 alcoholic drinks annually) |
|  | hypertension | Yes (self-reported hypertension; use of antihypertensive medication) |
|  | hypercholesterolemia | Yes (self-reported high cholesterol levels; use of cholesterol-lowering medications) |
|  | diabetes | Yes (diabetes was defined as self-reported diabetes mellitus; insulin or glucose-lowering medications; hemoglobin A1c (HbA1c) ≥ 6. 5%; and fasting blood glucose (FBG) ≥ 7.0 mmol/L.) |
|  | coronary heart disease (CHD) | Yes (self-reported as yes.) |
|  | stroke |  |
|  | chronic obstructive pulmonary disease  (COPD) |  |
|  | malignancy |  |

Reference: Cheng Q, Liu C, Zhong H, Wang Z, Zhou S, Sun J, Zhao S, Deng J: Comparison of systemic immunoinflammatory biomarkers for assessing severe abdominal aortic calcification among US adults aged≥40 years: A cross-sectional analysis from NHANES. PLoS One 2025, 20(6):e0325949.
