# Supplementary material for: Association between neutrophil to high-density lipoprotein cholesterol ratio and abdominal aortic calcification in US adults: A cross-sectional study
Source: Medicine (Baltimore). 2026 May 22;105(21):e49001. doi: 10.1097/MD.0000000000049001 (PMC13200935; doi:10.1097/MD.0000000000049001)
Supplement: Supplementary file 2 [file medi-105-e49001-s002.docx]

**S2 Table** Sensitivity analysis for the association between NHR, ACC score and severe AAC.

|  | After multiple imputation for missing data | | | |
| --- | --- | --- | --- | --- |
| Exposure | AAC Score | | Severe AAC | |
|  | β (95% CI) | *p* value | OR (95% CI) | *p* value |
| Crude model (Model 1) ^a^ | | | | |
| Continuous NHR | 0.103 (0.037, 0.169) | **0.002** | 1.098 (1.037, 1.161) | **0.001** |
| Categories NHR | | | |  |
| Tertile 1 | Refence | - | Refence | - |
| Tertile 2 | 0.390 (0.091, 0.689) | **0.011** | 1.395 (1.012, 1.933) | **0.043** |
| Tertile 3 | 0.704 (0.405, 1.003) | **<0.001** | 1.829 (1.346, 2.503) | **<0.001** |
| *P* for tend | 0.498 (0.287, 0.709) | **<0.001** | 1.533 (1.234, 1.913) | **<0.001** |
| Partially adjusted model (Model 2) ^b^ | | | | |
| Continuous NHR | 0.118 (0.057, 0.179) | **<0.001** | 1.136 (1.063, 1.211) | **<0.001** |
| Categories NHR | | | | |
| Tertile 1 | Refence | - | Refence | - |
| Tertile 2 | 0.408 (0.133, 0.684) | **0.004** | 1.379 (0.975, 1.957) | 0.070 |
| Tertile 3 | 0.749 (0.471, 1.028) | **<0.001** | 1.970 (1.410, 2.770) | **<0.001** |
| *P* for tend | 0.530 (0.333, 0.727) | **<0.001** | 1.615 (1.275, 2.055) | **<0.001** |
| Fully adjusted model (Model 3) ^c^ | | | | |
| Continuous NHR | 0.071 (0.006, 0.136) | **0.034** | 1.093 (1.014, 1.174) | **0.017** |
| Categories NHR | | | | |
| Tertile 1 | Refence | - | Refence | - |
| Tertile 2 | 0.338 (0.054, 0.622) | **0.020** | 1.278 (0.873, 1.876) | 0.207 |
| Tertile 3 | 0.617 (0.320, 0.915) | **<0.001** | 1.677 (1.144, 2.469) | **0.008** |
| *P* for tend | 0.436 (0.226, 0.647) | **<0.001** | 1.441 (1.100, 1.895) | **0.008** |

^a^Crude model (Model 1): no covariates were adjusted.

^b^Partially adjusted model (Model 2): adjusted for age and race.

^c^Fully adjusted model (Model 3): age, race, BMI, diabetes, hypercholesterolemia, hypertension, smoke, coronary heart disease, stroke, COPD, malignancy, serum phosphorus, total 25-hydroxyvitamin D and total cholesterol were adjusted.

CI: confidence interval, NHR: neutrophil to high-density lipoprotein cholesterol ratio, AAC: abdominal aortic calcification, BMI: body mass index, COPD: chronic obstructive pulmonary disease.
