# Supplementary material for: Association between neutrophil to high-density lipoprotein cholesterol ratio and abdominal aortic calcification in US adults: A cross-sectional study
Source: Medicine (Baltimore). 2026 May 22;105(21):e49001. doi: 10.1097/MD.0000000000049001 (PMC13200935; doi:10.1097/MD.0000000000049001)
Supplement: Supplementary file 3 [file medi-105-e49001-s003.docx]

**S3 Table** Sensitivity analysis for the association between NHR, ACC score and severe AAC.

|  | After removing the extreme values | | | |
| --- | --- | --- | --- | --- |
| Exposure | AAC Score | | Severe AAC | |
|  | β (95% CI) | *p* value | OR (95% CI) | *p* value |
| Crude model (Model 1) ^a^ | | | | |
| Continuous NHR | 0.180 (0.088, 0.272) | **<0.001** | 1.155 (1.056, 1.260) | **0.001** |
| Categories NHR | | | |  |
| Tertile 1 | Refence | - | Refence | - |
| Tertile 2 | 0.295 (-0.022, 0.611) | 0.068 | 1.259 (0.895, 1.776) | 0.187 |
| Tertile 3 | 0.750 (0.423, 1.076) | **<0.001** | 1.773 (1.277, 2.476) | **<0.001** |
| *P* for tend | 0.530 (0.299, 0.761) | **<0.001** | 1.499 (1.189, 1.898) | **<0.001** |
| Partially adjusted model (Model 2) ^b^ | | | | |
| Continuous NHR | 0.180 (0.094, 0.265) | **<0.001** | 1.160 (1.050, 1.280) | **0.003** |
| Categories NHR | | | | |
| Tertile 1 | Refence | - | Refence | - |
| Tertile 2 | 0.323 (0.031, 0.615) | **0.030** | 1.257 (0.870, 1.823) | 0.224 |
| Tertile 3 | 0.748 (0.444, 1.051) | **<0.001** | 1.774 (1.239, 2.554) | **0.002** |
| *P* for tend | 0.529 (0.314, 0.743) | **<0.001** | 1.500 (1.164, 1.941) | **0.002** |
| Fully adjusted model (Model 3) ^c^ | | | | |
| Continuous NHR | 0.142 (0.052, 0.232) | **0.002** | 1.123 (1.005, 1.254) | **0.039** |
| Categories NHR | | | | |
| Tertile 1 | Refence | - | Refence | - |
| Tertile 2 | 0.282 (-0.008, 0.573) | 0.057 | 1.240 (0.837, 1.843) | 0.285 |
| Tertile 3 | 0.632 (0.317, 0.947) | **<0.001** | 1.634 (1.095, 2.451) | **0.017** |
| *P* for tend | 0.447 (0.224, 0.670) | **<0.001** | 1.441 (1.100, 1.895) | **0.017** |

^a^Crude model (Model 1): no covariates were adjusted.

^b^Partially adjusted model (Model 2): adjusted for age and race.

^c^Fully adjusted model (Model 3): age, race, BMI, diabetes, hypercholesterolemia, hypertension, smoke, coronary heart disease, stroke, COPD, malignancy, serum phosphorus, total 25-hydroxyvitamin D and total cholesterol were adjusted.

CI: Confidence interval, NHR: Neutrophil to high-density lipoprotein cholesterol ratio, AAC: Abdominal aortic calcification, BMI: Body mass index, COPD: Chronic obstructive pulmonary disease.
