# Supplementary material for: Association between neutrophil to high-density lipoprotein cholesterol ratio and abdominal aortic calcification in US adults: A cross-sectional study
Source: Medicine (Baltimore). 2026 May 22;105(21):e49001. doi: 10.1097/MD.0000000000049001 (PMC13200935; doi:10.1097/MD.0000000000049001)
Supplement: Supplementary file 4 [file medi-105-e49001-s004.docx]

**S4 Table** Sensitivity analysis for the association between NHR, ACC score and severe AAC.

|  | When analyzing participants aged ≧60 years | | | |
| --- | --- | --- | --- | --- |
| Exposure | AAC Score | | Severe AAC | |
|  | β (95% CI) | *p* value | OR (95% CI) | *p* value |
| Crude model (Model 1) ^a^ | | | | |
| Continuous NHR | 0.249 (0.122, 0.376) | **<0.001** | 1.129 (1.055, 1.208) | **<0.001** |
| Categories NHR | | | |  |
| Tertile 1 | Refence | - | Refence | - |
| Tertile 2 | 0.663 (0.086, 1.240) | **0.024** | 1.364 (0.948, 1.969) | 0.096 |
| Tertile 3 | 1.473 (0.897, 2.050) | **<0.001** | 1.995 (1.415, 2.835) | **<0.001** |
| *P* for tend | 1.042 (0.634, 1.450) | **<0.001** | 1.630 (1.278, 2.089) | **<0.001** |
| Partially adjusted model (Model 2) ^b^ | | | | |
| Continuous NHR | 0.225 (0.105, 0.345) | **<0.001** | 1.137 (1.057, 1.222) | **<0.001** |
| Categories NHR | | | | |
| Tertile 1 | Refence | - | Refence | - |
| Tertile 2 | 0.500 (-0.046, 1.047) | 0.073 | 1.253 (0.853, 1.844) | 0.252 |
| Tertile 3 | 1.295 (0.747, 1.843) | **<0.001** | 1.894 (1.314, 2.747) | **<0.001** |
| *P* for tend | 0.916 (0.528, 1.303) | **<0.001** | 1.571 (1.213, 2.043) | **<0.001** |
| Fully adjusted model (Model 3) ^c^ | | | | |
| Continuous NHR | 0.132 (0.006, 0.258) | **0.040** | 1.101 (1.013, 1.192) | **0.020** |
| Categories NHR | | | | |
| Tertile 1 | Refence | - | Refence | - |
| Tertile 2 | 0.306 (-0.241, 0.853) | 0.273 | 1.178 (0.777, 1.791) | 0.441 |
| Tertile 3 | 0.943 (0.360, 1.526) | **0.002** | 1.637 (1.076, 2.503) | **0.022** |
| *P* for tend | 0.667 (0.255, 1.079) | **0.002** | 1.417 (1.053, 1.913) | **0.022** |

^a^Crude model (Model 1): no covariates were adjusted.

^b^Partially adjusted model (Model 2): adjusted for age and race.

^c^Fully adjusted model (Model 3): age, race, BMI, diabetes, hypercholesterolemia, hypertension, smoke, coronary heart disease, stroke, COPD, malignancy, serum phosphorus, total 25-hydroxyvitamin D and total cholesterol were adjusted.

CI: Confidence interval, NHR: Neutrophil to high-density lipoprotein cholesterol ratio, AAC: Abdominal aortic calcification, BMI: Body mass index, COPD: Chronic obstructive pulmonary disease.
